# Neutrophil heterogeneity in complement C1q expression associated with sepsis mortality

**DOI:** 10.3389/fimmu.2022.965305

**Published:** 2022-08-02

**Authors:** Alissa Trzeciak, Raj Kumar Mongre, Ma Rie Kim, Kihong Lim, Rafael A. Madero, Christopher N. Parkhurst, Anthony P. Pietropaoli, Minsoo Kim

**Affiliations:** ^1^ Department of Microbiology and Immunology, David H. Smith Center for Vaccine Biology and Immunology, University of Rochester, Rochester, NY, United States; ^2^ Department of Biomedical Engineering, University of Rochester Medical Center, Rochester, NY, United States; ^3^ Division of Pulmonary and Critical Care Medicine, Weill-Cornell Medicine, New York, NY, United States; ^4^ Pulmonary and Critical Care Medicine Division, University of Rochester, Rochester, NY, United States

**Keywords:** neutrophil, sepsis, efferocytosis, C1q, inflammation

## Abstract

Sepsis is a life-threatening systemic inflammatory condition causing approximately 11 million annual deaths worldwide. Although key hyperinflammation-based organ dysfunctions that drive disease pathology have been recognized, our understanding of the factors that predispose patients to septic mortality is limited. Due to the lack of reliable prognostic measures, the development of appropriate clinical management that improves patient survival remains challenging. Here, we discovered that a subpopulation of CD49c^high^ neutrophils with dramatic upregulation of the complement component 1q (C1q) gene expression arises during severe sepsis. We further found that deceased septic patients failed to maintain C1q protein expression in their neutrophils, whereas septic survivors expressed higher levels of C1q. In mouse sepsis models, blocking C1q with neutralizing antibodies or conditionally knocking out C1q in neutrophils led to a significant increase in septic mortality. Apoptotic neutrophils release C1q to control their own clearance in critically injured organs during sepsis; thus, treatment of septic mice with C1q drastically increased survival. These results suggest that neutrophil C1q is a reliable prognostic biomarker of septic mortality and a potential novel therapeutic target for the treatment of sepsis.

## Introduction

Sepsis is defined as a systemic inflammatory response to infection that causes vital organ dysfunction. Although sepsis is one of the most common reasons for hospital death (1, 2), patient responses to this disease are highly heterogeneous (3) making it difficult to identify the critical pathophysiologic processes that lead to sepsis fatality (2, 4). Thus, optimal improvements in the development of sepsis therapies require precise prognostic and predictive measures to identify unique subgroups of patients at high risk of poor outcomes that may benefit from early appropriate matching with resources to provide aggressive and targeted adjunctive interventions (5, 6).

Organ failure is the ultimate threat to the patient survival in sepsis. Organ failure results from the dysregulated host response to infection, not from the infection itself. Examination of autopsy samples from patients with multiple organ failure reveals massive neutrophil accumulation along blood vessels and within tissues (7, 8). Once they complete their action, early infiltrated neutrophils should be quickly cleared from infected tissue sites. In non-resolving inflammation, neutrophils often persist at the inflamed site as a result of impaired neutrophil clearance (9–13). Delayed neutrophil clearance is often associated with widespread tissue damage, organ failure, and ultimately death in a wide range of inflammatory diseases (14–18). The cellular and molecular signals that drive the initiation of neutrophil-mediated inflammatory responses are well studied, but we have a relatively poor understanding of the mechanisms through which the neutrophil response is resolved.

Here, we found that the expression level of complement component C1q in neutrophils determines a subpopulation of patients who have more severe immune dysregulation and thus higher mortality. Furthermore, our animal studies have shown that neutrophil-derived C1q is necessary for septic survival, and that treatment of septic mice with C1q drastically increases the survival. Therefore, a favorable sepsis prognosis depends on neutrophil specific C1q expression and patients whose neutrophils are unable to produce the C1q protein have a higher likelihood of sepsis mortality.

## Results

### Identification of a neutrophil population arising in septic patients with a poor outcome

**Figure 1:**
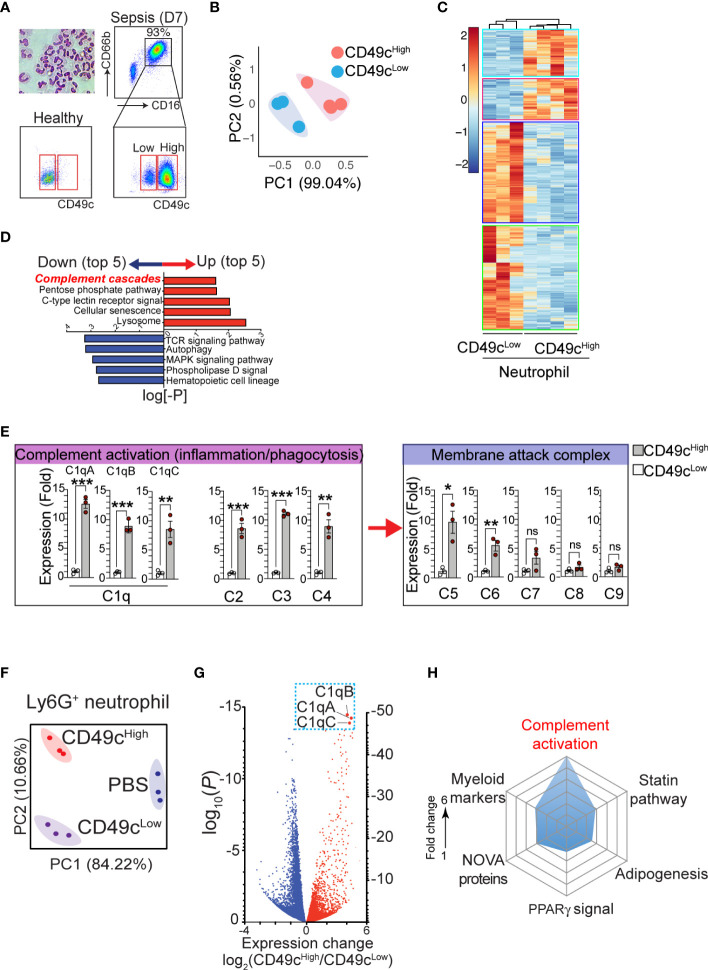
The phenotypic heterogeneity in neutrophils from septic patients. **(A)**, Isolated neutrophils from the blood were stained with hematoxylin and eosin after cytospin and confirmed > 99.5% of pure neutrophils by morphology with a multilobed nucleus, without other myeloid cell types such as monocyte and macrophage. The additional gating strategy for CD49c^high^ and CD49c^low^ neutrophil populations in the patient blood is also shown. The pseudo color plots demonstrate CD49c upregulation on septic neutrophils (day 7) compared with neutrophils from a healthy donor. **(B)** PCA of differentially expressed genes in neutrophil subsets (CD49c^high^ vs. CD49c^low^) from septic patients. **(C)**, Heatmap of RNA-sequencing data from CD49c^high^ (n = 4) versus CD49c^low^ (n = 3) neutrophils sorted from septic patient blood samples. Heatmap represents top 1082 genes chosen from highest 0.25% p-value summary. **(D)**, Pathway analysis reveals a distinguishable pattern of gene-program changes in neutrophils expressing higher levels of CD49c. Bidirectional plot represents the likelihood (p>0.05) that signaling pathways were enriched (*red*) or reduced (*blue*) in CD49c^high^ vs>. CD49c^low^ septic patient neutrophils. **(E)**, Expression of the indicated genes in septic neutrophils by qRT-PCR. **(F)**, PCA of differentially expressed genes in neutrophil subsets in endotoxemic mice or naïve (PBS) mice. **(G)**, Volcano plot shows comparison of differentially expressed genes in septic neutrophil subsets. Red denotes genes increased and blue denotes genes decreased in neutrophils. **(H)**, Spider plot using Enrichr GO database showing the top six categories of signaling pathways relating to genes upregulated in CD49c^high^ versus CD49c^low^ septic neutrophils. Measured as a function of fold change of CD49c high over low for likelihood of genes falling into the listed categories. Data are presented as mean ± SEM. Data were analyzed by ordinary one- way ANOVA with Tukey’s multiple comparison post-test for *b-e* (*P < 0.05) or nonparametric Mann-Whitney test for i (**P < 0.01, ***P < 0.001).

Previously, we reported that the expression of neutrophil CD49c correlated with sepsis diagnosis (19). Examination of neutrophils isolated from septic patients further revealed that two distinct subpopulations of neutrophils with higher and lower CD49c expression (CD66b^+^CD16^+^CD49c^high^ and CD66b^+^CD16^+^CD49c^low^) arise during sepsis (**Figure 1A**). Identifying the heterogeneity in septic neutrophils with differential CD49c expression prompted further investigations to discover novel genetical and/or functional classifiers in the neutrophil populations. First, we sought to define the transcriptional signatures of CD49c^high^ vs. CD49c^low^ neutrophils isolated from septic patients by RNAseq. We confirmed that there was substantial differential gene expression between neutrophils isolated from healthy donors and those isolated from sepsis patients (**Figures S1A, B**). Principal component analysis (PCA) of the differentially expressed genes identified in septic neutrophils revealed clear transcriptional disparities between CD49c^high^ and CD49c^low^ neutrophils from patients (**Figure 1B**). Among the 56,871 genes identified, 1,082 were differentially expressed in CD49c^high^ neutrophils (**Figure 1C**). Pathway analysis revealed that sepsis led to a significant upregulation of genes related to the innate inflammatory response in CD49c^high^ neutrophils (**Figure 1D**). Interestingly, among the top five innate inflammatory response gene groups, we detected the robust upregulation of complement pathway associated genes. Of those, we further validated upregulation of several key complement functional genes by qRT-PCR (**Figure 1E**). Note that the most significant changes were detected in the complement recognition and activation step, including genes encoding C1q proteins, but not in the genes involved in the terminal pathway for the membrane attack complex (MAC).

To further verify the gene expression results in a controlled mouse model, neutrophils from endotoxemic mice were FACS-sorted into CD49c^high^ and CD49c^low^ neutrophil populations, and mRNA was analyzed by RNAseq. PCA plots (**Figure 1F**) showed clear transcriptional separation of the CD49c subsets in mice, and a volcano plot showed differences among specific genes (**Figure 1G**). Among the 16,384 genes identified, 5,717 were found to be differentially expressed between CD49c^high^ and CD49c^low^ septic neutrophils (**Figure S1C**). Of these genes, we identified 326 genes that were significantly upregulated in CD49c^high^ neutrophils compared to CD49c^low^ neutrophils. Using the Enrichr online database for analysis (20), we ranked these genes according to categories that best described their protein function. Importantly, we found that CD49c^high^ expression was highly correlated with genes in the complement cascade (**Figure 1H**). Furthermore, the most differentially expressed complement cascade associated genes were *C1qa*, *C1qb*, and *C1qc* genes, which encode the C1qA, C1qB, and C1qC chains that assemble into the complement factor C1q (**Figure 1G**) (21), thus confirming our observations in septic patient samples (**Figures 1D, E**).

### Loss of C1q expression in neutrophils is associated with sepsis mortality

**Figure 2:**
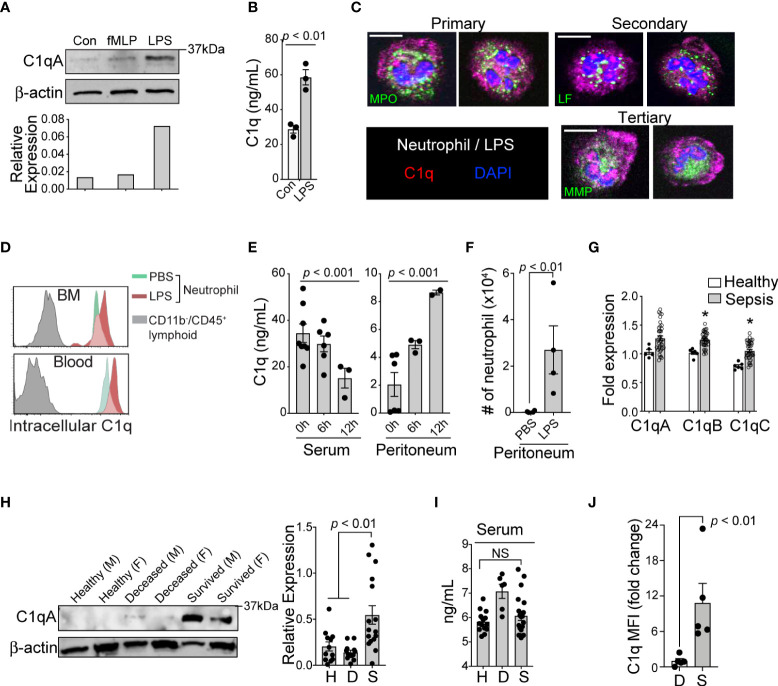
Loss of C1q expression represents the functional heterogeneity of neutrophil that are associated with sepsis mortality. **(A)**, *Top:* Western blot analysis of C1q expression in healthy human neutrophils after stimulation with fMLP (10 μM) or LPS (10 μg/mL) for 1h (representative of three subjects), *bottom:* densitometry quantification. **(B)**, Total C1q secretion of healthy human neutrophils stimulated with LPS (10 μg/mL) for 1h *in vitro*. **(C)**, Immunofluorescence image of human neutrophils stained with Abs against C1q (red), myeloperoxidase (MPO, green), lactoferrin (LF, green), and MMP-9 (MMP, green). Scale bar, 10 μm. **(D)**, Representative MFI histograms of C1q expression from the bone marrow (BM) and circulation of CD45^+^/CD11b^+^/Ly6G/C^+^ neutrophils (*green* peaks = PBS, *red* peaks = LPS) versus CD45^+^/CD11b^-^ lymphoid cells (*grey* peaks). **(E)**, Total C1q secretion in the serum (*left*) and peritoneum (*right*) during acute systemic inflammation at 6 and 12h as measured by ELISA. **(F)**, Absolute neutrophil counts from peritoneal lavage isolates in PBS vs LPS treated mice (n = 4 mice each). **(G)**, qPCR of C1qa, C1qb, and C1qc in healthy subjects (n = 5-6) and septic patients (n=22) as compared to three averaged housekeeping genes. **(H)**, *Left*, C1q (weight = 29Da) western blot analysis on neutrophils isolated from healthy (H), deceased (D), and survived (S) sepsis patients. M, male; F, female; Data representative of six experiments. *Right*, quantification of total C1q protein expression by western blot in healthy (H), deceased (D), and survived (S) patient neutrophils (n = 12, 12, and 16, respectively). **(I)**, Total serum C1q of healthy (H), deceased (D), and survived (S) patients measured by ELISA (n = 14, 6, and 20, respectively). NS, Not significant. **(J)**, C1q levels in neutrophils from the peritoneal lavage of septic mice were analyzed by flow cytometry (n = 4 mice/group). Data are presented as mean ± SEM. Data were analyzed by ordinary one-way ANOVA with Tukey’s multiple comparison post-test in **(E, H, I)**, by students t-test for **(B, J)**, and by Welch’s t-test for two-group comparison for **(G)**.

To confirm the expression of C1q protein in human neutrophils, we stimulated neutrophils with lipopolysaccharide (LPS) and N-formylmethionine-leucyl-phenylalanine (fMLP) and found that LPS alone significantly elevated both the protein expression (**Figure 2A**) and secreted levels of C1q *in vitro* (**Figure 2B**). Immunohistologic examination of human neutrophils suggested that C1q was stored within organelles lacking myeloperoxidase (primary granules), lactoferrin (secondary granules), and MMP-9 (tertiary granules); these organelles might be secretory vesicles (**Figure 2C**). Consistent with human neutrophils, flow cytometry analysis also confirmed the significant expression of C1q in neutrophils from both naïve mice and LPS-treated mice (**Figure 2D**). To understand how C1q protein levels change in various tissues over time, we induced endotoxemia in mice by intraperitoneal injection of LPS and found that while the serum levels of C1q significantly declined during the first 6-12 h after LPS injection, C1q levels at the peritoneal site of inflammation increased significantly (**Figure 2E**). Notably, the elevated local C1q level coincided with massive infiltration of neutrophils into the peritoneum (**Figure 2F**), suggesting that newly recruited neutrophils may be a main source of tissue C1q during inflammation.

In neutrophils isolated from the peripheral blood of septic patients, quantitative reverse-transcription PCR (qRT-PCR) analyses revealed that genes associated with C1q expression were significantly increased, irrespective of the survival status of the individual (**Figure 2G**). Strikingly, only neutrophils from survived sepsis patients but not from patients who died were able to translate and maintain intracellular C1q (**Figure 2H**). This result indicates that poor patient outcomes were associated with the loss of C1q protein, while all sepsis patients retained the ability to express *C1q* transcripts. Importantly, we did not observe any significant differences in serum C1q levels between healthy subjects and patient groups (**Figure 2I**). As in human patients, neutrophils from survived septic mice but not from mice that died were able to translate and maintain intracellular C1q (**Figure 2J**).

### Neutrophil C1q is essential for sepsis survival

**Figure 3:**
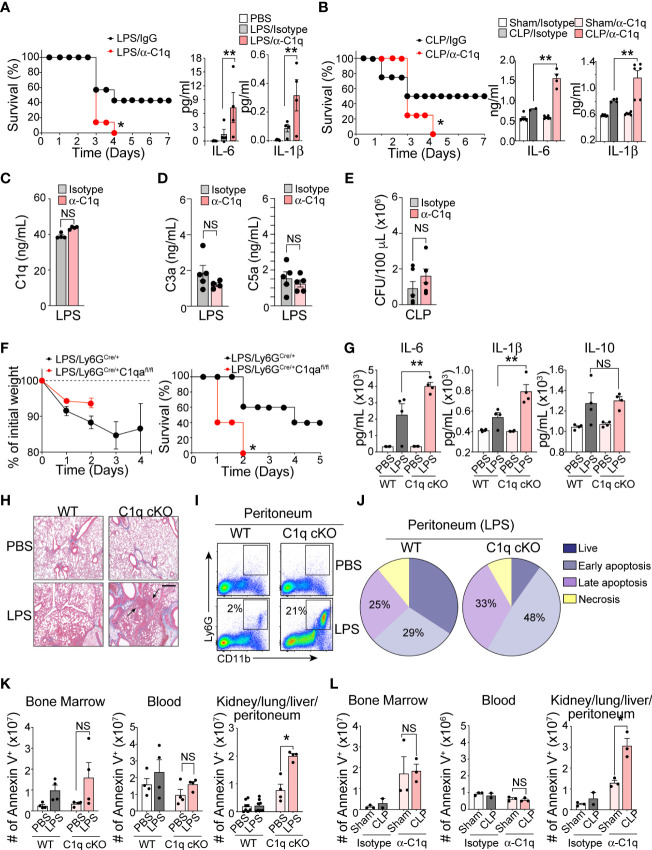
Neutrophil C1q is essential for sepsis survival. **(A)**, *Left:* Survival assay of LPS-induced endotoxemia mice treated intraperitoneally (IP) with isotype IgG control antibody or anti-C1q neutralizing antibody (10 μg/mouse) one hour prior to IP injection of LPS. n = 6 per group. *Right:* IL-6 and IL-1β secretion in the serum 6 hours post-sepsis induction, data representative of three separate experiments. **(B)**, *Left:* Survival assay of CLP-induced sepsis in WT mice treated IP with isotype IgG control antibody or anti-C1q neutralizing antibody (10 μg/mouse) one hour prior to surgeries. n = 6 per group. *Right:* IL-6 and IL-1β secretion in the serum of Sham or CLP WT mice treated with either isotype control IgG antibody or anti-C1q neutralizing antibody one hour prior to surgeries. ELISA was measured 24 hours post-sepsis induction, data pooled from two experiments. **(C)**, Total C1q secretion in the serum of WT mice IP-treated with 15 mg/kg of LPS and either isotype control IgG antibody or anti-C1q neutralizing antibody 1 hour prior to LPS injection. ELISA measured 6 hours post-sepsis induction, n = 6 per group. **(D)**. Quantification of the serum C3a and C5a in LPS-treated mice using ELISA (n = 5 mice/group). **(E)** Bacterial loads in the blood of septic mice were measured at 48 h of post-CLP. The bacterial colonies grown on the blood agar plates were calculated as CFU per mL of blood (n = 5 mice in each group). **(F)**. Sepsis severity (*left*) and survival curves (*right*) of Ly6G^Cre/+^:C1qa^+/+^ (WT) or Ly6G^Cre/+^:C1qa^fl/fl^ mice (C1q cKO) mice treated with 15mg/kg LPS, n = 5 mice per group. **(G)**, IL-6, IL-1β, and IL-10 secretion in the serum of WT or C1q cKO mice treated with PBS (white bars = WT, light pink bars = cKO) or LPS (grey bars = WT, dark red bars = cKO), n = 4 per group. **(H)**, Histological analysis of the inflamed lung in Ly6G^Cre/+^C1qa^+/+^ vs. Ly6g^Cre/+^C1qa^fl/fl^ mice 24 hours after PBS or 18 mg/kg of IP LPS injection using Masson’s trichrome staining. White arrows indicate blood vessels. Scale bars, 40 μm. **(I)**, Typical flow cytometry profile of neutrophils in the peritoneum during acute LPS stimulation for 36 h (n = 3–4 mice per group). **(J)**, The pie charts depict the proportion of live, necrotic (PI^+^ and Annexin^−^), and early (PI^−^ and Annexin^+^) and late (PI^+^ and Annexin^+^) apoptotic populations in each group. Data are presented as mean of n = 3. **(K)**, Number of apoptotic neutrophils accumulated in the bone marrow, blood, and peripheral organs (kidneys, lungs, liver, peritoneum pooled) measured in absolute number of Ly6G^+^/annexin V^+^ cells. WT vs C1q cKO treated with PBS or LPS (n = 4 per group). **(L)**, Number of apoptotic neutrophils in Sham surgery vs. CLP mice with IgG isotype control or anti-C1q antibody (n = 3 mice per group). Data are presented as mean ± SEM. Data were analyzed by Mantel-Cox for **(A, B, F)**, ordinary one-way ANOVA with Tukey’s multiple comparison post-test or two-way ANOVA in **(A–E, K, L)**. (*P < 0.05; **P < 0.01). NS, not significant.

C1q is the initiator molecule in the classical complement cascade, which ultimately leads to cell lysis *via* the formation of the MAC (21). The presence of high C1q levels in inflamed tissue and increased neutrophil C1q production in sepsis survivors led us to hypothesize that C1q has a role outside of the classical complement cascade, functioning as an important inflammatory mediator during severe systemic inflammation. We further predicted that secretion of C1q locally at inflamed tissue sites by newly recruited neutrophils may be critical for patient survival during sepsis. To test this hypothesis, we administered a C1q neutralizing antibody (22) into the mouse peritoneal cavity 1 h prior to septic challenge. For this functional assay, we used the following two mouse models: LPS-induced endotoxemia, which can bypass the need for C1q to bind to antibody-coated bacteria and initiate the complement cascade, and cecal ligation and puncture (CLP) surgery to induce clinically relevant polymicrobial sepsis. Blocking C1q function locally at the site of inflammation dramatically increased sepsis mortality and significantly increased the serum levels of the proinflammatory cytokines IL-6 and IL-1b in both the endotoxemia and CLP mouse sepsis models (**Figures 3A, B**). Importantly, local injection of the anti-C1q neutralizing antibody did not alter the systemic level of C1q and other complement components in the serum (**Figures 3C, D**) and did not change local bacterial clearance (**Figure 3E**).

Determining the contributions of C1q to immune functions *in vivo* is challenging due to the increased mortality of *C1qa* subunit-knockout mice, which succumb to severe autoimmunity resulting from impaired clearance of apoptotic cells (23). To circumvent this problem and further assess the function of neutrophil-derived C1q in sepsis, we generated a conditional knockout C1q^flox/flox^; Ly6G^Cre/+^ (C1q cKO) mouse strain by crossing C1qa-floxed mice with a mouse line in which the first exon of the *Ly6g* gene is replaced by a knock-in allele encoding Cre recombinase (24). Deletion of the floxed *C1qa* subunit alleles and the absence of protein expression were confirmed by PCR and western blot analysis (**Figures S2A, B**). We used C1q^wt/wt^; Ly6G^Cre/+^ (WT) mice as littermate controls. Neutrophil development, bacterial clearance, TLR4 surface expression, and inflammation-mediated oxidative burst by neutrophils isolated from C1q cKO mice were unaffected (**Figures S2C–F**). Although C1q cKO mice showed disease severity similar to that of their WT littermates during the early period of mild endotoxemia, the absence of neutrophil-derived C1q resulted in dramatically increased mortality after LPS treatment (**Figure 3F**). The serum levels of IL-6 and IL-1β were significantly elevated in C1q cKO mice compared with control mice, indicating an increased sepsis severity (**Figure 3G**). In addition, lung histology pointed to an increased focal interstitial thickening and perivascular inflammation in C1q cKO lungs compared to WT lungs after LPS treatment (**Figure 3H**). Flow cytometry analysis further revealed that C1q cKO mice maintained a significantly higher proportion of neutrophils (CD11b^high^Ly6G^high^) in the inflamed peritoneum during LPS induced endotoxemia (**Figure 3I**). This finding could be directly attributed to a decreased rate of apoptotic neutrophil clearance (or “efferocytosis”) in C1q cKO mice as compared to WT littermates (**Figure 3J**). Indeed, C1q cKO mice and C1q neutralizing antibody-treated mice showed the accumulation and/or delayed removal of apoptotic neutrophils in the inflamed lungs and the peritoneal space, as measured within 24 hr of endotoxemia and 48 hr of CLP (**Figures 3K, L**).

### Neutrophil C1q is essential for resolution of inflammation

**Figure 4:**
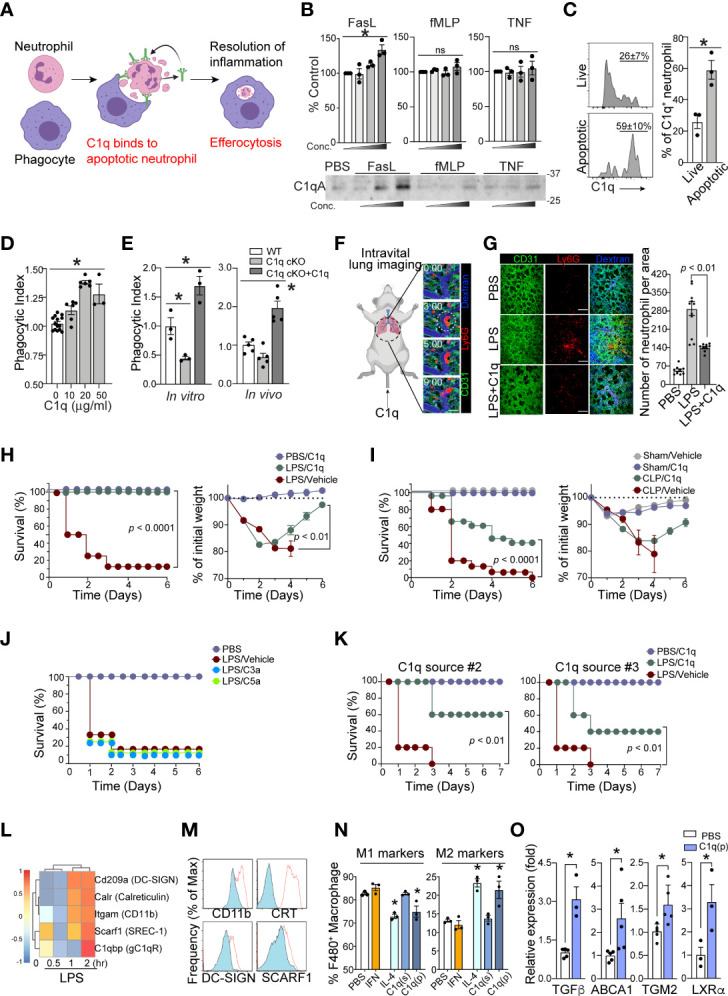
Neutrophil C1q is essential for resolution of inflammation. **(A)**, Neutrophil-specific C1q expression is essential for proper efferocytosis of apoptotic neutrophils by local resident macrophage, leading to resolution of infection and survival. Without C1q, apoptotic neutrophils build up causing prolonged inflammation and therefore poorer prognosis and eventually, death. **(B)**, C1q secretion by neutrophils in response to FasL (0.2, 1, 5 μg/ml), fMLP (0.2, 1, 5 μM), or TNF-α (0.1, 1, 10 μg/ml) stimulation was determined by Western blot analysis of neutrophil supernatants with an C1q-specific antibody. *Upper:* densitometry quantification. *Bottom:* Each panel shows one representative image of three replicated experiments. **(C)**, *Left*: MFIs of surface bound C1q on Annexin V^-^ live (*top*) and Annexin V^+^ apoptotic (*bottom*) human neutrophils treated with C1q. *Right*: Quantification of C1q expression as a percentage of total neutrophils on live and apoptotic cells (n = 3 per group). **(D)**, An increase in C1q concentration results in a similar increase in human apoptotic neutrophil clearance by a PMA-differentiated U937 monocyte cell line. n = 3-6. Data pooled from five experiments. **(E)**, C1q cKO neutrophils exhibit impaired clearance by peritoneal macrophages, which is rescued by the addition of 10 μg of soluble C1q (n=3 per group). **(F)**, Representative real-time imaging of capillary obstruction with Ly6G^+^ cells (red) in sepsis-induced acute lung injury model (**Supplementary Video 3**). The capillary flow (dextran, blue) and blood vessel (anti-CD31 antibody, green) are shown. White circle indicates newly entrapped neutrophil aggregates obstructing the capillary. Scale bars, 20 μm. **(G)**, Representative intravital imaging of the pulmonary microcirculation in the PBS, LPS, and LPS+C1q group. Scale bars, 100 μm in magnified. *Right:* Comparisons of Ly6G^+^ cell count among PBS, LPS, LPS+C1q groups. n = 3 – 4 mice per group. 3 – 4 fields of view per mouse, one-way ANOVA with *post hoc* Holm–Sidak’s multiple comparisons test. Data are presented as mean ± SEM. **(H–J)**, Survival curves (*left*) and sepsis severity (*right*) of WT mice treated with LPS **(H, J)**, or CLP **(I)** that received 20 μg of C1q, C3a, C5a, or vehicle 30 min prior to sepsis induction and again at 6h and 24h post sepsis induction. n = 3- 4 for PBS and n = 8 – 14 for per treatment group. **(K)**, Survival curves (*left*) and sepsis severity (*right*) of WT mice treated with LPS that received 20 μg of C3a or C5a 30 min prior to sepsis induction and again at 6h and 24h post sepsis induction. n = 7 per group. **(L)**, LPS-stimulated bone marrow derived macrophages exhibit a robust elevation in C1q receptor genes within 2h of activation (GEO dataset: GSE116220). **(M)**, MFI surface expression of C1qRs CD11b (*Itgam*), Calreticulin (CRT, *Calr*), DC-SIGN (*Cd209a*), and SCARF-1 (*Srec-1*) on peritoneal macrophages stimulated with LPS (red). n =3 **(N)**, Peritoneal macrophages were stimulated *in vitro* with either 40 ng/ml IFN-γ, 1 μg/ml IL-4, 10 μg/ml soluble C1q(s), or 0.1 μg/ml plate-bound C1q (p) to induce M1 pro-inflammatory vs. M2 pro-resolving polarization. The frequency of M1 markers (*left*) was measured as the percentage of CD45^+^/CD11b^+^/F4/80^+^ cells that were CD206^low^/MerTK^low^/CD86^high^ while M2 markers (*right*) were CD206^high^/MerTK^high^/CD86^low^, n = 3 per condition, data representative of three separate experiments. **(O)**, Expression of the indicated genes in peritoneal macrophages stimulated *in vitro* with 0.1 μg/ml plate-bound C1q (P) was measured by qRT-PCR. Data are presented as mean ± SEM. Data were analyzed by ordinary one-way ANOVA with Tukey’s multiple comparison post-test in **(B, E, N, O)**, students t-test in **(C)**, Mantel-Cox and two-way ANOVA for **(H–K)**. (*P < 0.05), and by Welch’s one-way test for multiple group comparison for **(D)**.

C1q binds to phosphatidylserine (PS) on apoptotic cells and mediates efferocytosis (25, 26). Therefore, we hypothesized that local C1q secretion by apoptotic neutrophils is necessary for prompt neutrophil efferocytosis and removal by phagocytes, which is an essential response to achieve a relatively good patient prognosis during sepsis (**Figure 4A**). To investigate whether neutrophils secrete C1q in response to inflammatory stimuli, we first performed western blot analysis of neutrophil supernatants. Interestingly, even with high concentrations of inflammatory stimuli that induce neutrophil degranulation, such as TNFα or fMLP, we failed to detect significant release of soluble C1q from neutrophils (**Figure 4B**). To screen for potential stimulators that trigger C1q release from neutrophils, we next turned our attention to neutrophil apoptosis and found that the secretion of C1q was most significantly enhanced after the induction of neutrophil apoptosis with Fas ligand (FasL) (**Figure 4B**).

Neutrophil clearance is crucial for the resolution of septic inflammation and is therefore critical for patient survival. If the secretion of C1q from neutrophils depends on apoptosis, conceptually, it may be possible that neutrophil-derived C1q functions as an important “eat me” signal to promote efferocytosis by local phagocytes (27). Flow cytometry analyses of live vs. apoptotic human neutrophils further revealed a greater extent of C1q binding on the surface of apoptotic neutrophils (**Figure 4C**). In addition, C1q produced significant dose-dependent enhancement of the phagocytic uptake of apoptotic neutrophils by macrophages (**Figure 4D**). Consistently, neutrophils isolated from C1q cKO mice showed a marked decrease in phagocytic clearance by macrophages compared to WT neutrophils, which was reversed by the addition of exogenous C1q during the experiments (**Figure 4E**). Importantly, loss of intrinsic C1q expression in C1q cKO mice did not alter the ability of C1q cKO neutrophils to undergo apoptosis (**Figures S3A, B**).

Prior work utilizing a mouse model of sepsis-induced acute lung injury (ALI) demonstrated that massive tissue infiltration and subsequent sequestration of unresolved neutrophils in the pulmonary microcirculation led to acute respiratory distress syndrome (ARDS) (28). Indeed, intravital microscopy of the mouse lung with a custom-made pulmonary imaging window revealed a significant number of neutrophil aggregates in the pulmonary microcirculation of LPS-induced ALI mice (**Figure 4F** and **Suppl. Video 1**-**3**). Notably, unresolved neutrophil aggregates were a main cause of pulmonary capillary obstruction in sepsis-induced ALI mice (28). In LPS-induced ALI mice, administration of C1q significantly reduced the number of neutrophil aggregates in mice 24 hrs after LPS stimulation (**Figure 4G** and **Suppl. Video 4**). These findings parallel mouse survival assays, in which periodic injections of C1q, but not C3a or C5a, protected mice from sepsis lethality in both the LPS-induced endotoxemia model and the CLP surgery model of sepsis (**Figures 4H–J**). These results were further corroborated using serum purified C1q obtained from two independent commercial sources (**Figure 4K**).

After clearing apoptotic neutrophils, tissue-resident macrophages often initiate pro-resolution program by releasing anti-inflammatory and tissue-repairing cytokines, such as TGFβ and IL-10 (29, 30). Therefore, the expression of C1q by septic neutrophils that promotes phagocytosis by tissue-resident macrophages may directly influence the polarization of macrophages from a proinflammatory (M1) phenotype to an anti-inflammatory (M2) phenotype. Among the potential C1q receptors known to be expressed (**Figure 4L**), peritoneal macrophages isolated from LPS-treated mice upregulated at least four of the receptors (**Figure 4M**), suggesting that the apoptotic neutrophil-macrophage interactions during sepsis are at least in part mediated by these receptors. Indeed, plate-bound C1q (p) but not soluble C1q (s) significantly increased the expression of M2 markers on peritoneal macrophages while simultaneously suppressing the expression of M1 markers (**Figure 4N**). Therefore, our data suggest that neutrophil-derived C1q mediates the polarization of M2 macrophages and increases host survival by promoting tissue repair within local microenvironments. In addition, plate-bound C1q also enhanced the expression of several key resolution markers in macrophages (**Figure 4O**).

## Discussion

We discovered the presence of subpopulations of bloodstream neutrophils that express higher and lower levels of CD49c in ICU patients and experimental animals with sepsis. CD49c^high^ and CD49c^low^ neutrophils from septic patients are transcriptionally distinct, representing the heterogeneity among neutrophils in critically ill patients. Among those differences, the expression level of C1q in neutrophils determines a subpopulation of patients who have more severe immune dysregulation and thus higher mortality. Our study further demonstrates that the acute and drastic increase in neutrophils released from the bone marrow during sepsis and their contribution to the pool of C1q in local tissue microenvironments are critical for neutrophil clearance and inflammation resolution (31) and suggests that rebalancing defective neutrophil C1q production during sepsis, possibly by exogenous augmentation, may function as a novel sepsis therapy.

Genetic abnormalities resulting in C1q absence or dysfunction lead to a lupus-like autoimmune phenotype marked by increases in glomerular damage and neuropsychiatric disorders (20, 23, 32–35). In the context of C1q immunodeficiency, the accumulation of dying cells due to the lack of normal C1q functions promotes autoimmunity *via* autoantigens targeted to the surface of apoptotic cells (36). The survival of septic patients is critically dependent on the ability to both control the infection and regain homeostasis *via* resolution of the inflammatory responses. Consistent with the pathology associated with lupus-like phenotypes in humans, our data support the idea that neutrophils are an essential source of C1q in inflamed tissues and that loss of neutrophil-derived C1q results in impaired clearance of apoptotic cells during sepsis. Neutrophil efferocytosis is a process that facilitates the resolution of infection and inflammation by removing dead cells. In addition, tissue-resident macrophages that phagocytose dying cells often initiate a feed-forward resolution program of inflammation by upregulating anti-inflammatory and tissue-repairing mediators (37–39). Thus, C1q plays a key role during the resolution phase by functioning as an important “eat me” signal (40–42) and further promotes recovery and survival in septic patients.

Sources of C1q vary depending on the inflammatory phenotype and anatomical location. For instance, microglia are known to be the main producers of C1q in the brain (43), while endothelial cells are an essential source of C1q in the liver (44). While macrophages and liver endothelial cells are still the main sources of complement components during inflammation (44, 45), the production of C1q by these cells may be insufficient to compensate for the loss of C1q production by neutrophils at tissue sites during severe sepsis. Our study further demonstrates that the acute and drastic increase in neutrophils released from the bone marrow during sepsis and their contribution to the pool of C1q in local tissue microenvironments are critical for neutrophil clearance and inflammation resolution (31) and suggests that rebalancing defective neutrophil C1q production during sepsis, possibly by exogenous augmentation, may function as a novel sepsis therapy. In addition, measuring C1q expression in neutrophils may serve as a powerful diagnostic tool that can be exploited to more accurately assign sepsis endotypes and provide earlier treatment of acute systemic inflammation to increase the potential for survival in severely ill patients.

We previously showed that both CD49c^high^ and CD49c^low^ neutrophils express a similar level of CD11b, a well-known cell surface activation marker described (19). This suggests that the presence of a CD49c^high^ subpopulation, and the expression and secretion of intracellular C1q are not entirely dependent on neutrophil activation. Our finding leaves an unanswered fundamental question. Why do some sepsis patients express a high level of C1q in neutrophils, while others fail to maintain appropriate C1q expression? Although the full spectrum of neutrophil properties regarding C1q expression remains unexplored, it is likely that neutrophil heterogeneity may arise in the circulation, in which they acquire distinct phenotypic, transcriptional, and functional properties, that may later impact C1q production and host response to severe inflammation. Importantly, the microbiome has recently emerged as a key regulator of the neutrophil heterogeneity (46) and previous studies reported the presence of the microbiome in healthy human blood (47–50). Therefore, it is possible that the heterogeneous upregulation of C1q during sepsis may be associated with pre-priming of a subpopulation of circulating neutrophils by microbiomes in homeostasis. This may be an important topic for further studies in the future aimed at understanding the mechanisms of the microbiome-neutrophil interaction in homeostasis and potential relationships with C1q production during sepsis.

## Methods

### Animals

Adult male C57BL/6J mice (between 7 and 8 weeks of age) were purchased from the Jackson Laboratory and transferred to our facility, where they were allowed to acclimate for 7 days prior to sepsis induction. Ly6G^Cre/Cre^ homozygote males and females were a generous gift from Dr. Matthias Gunzer (University of Duisburg Essen) (24). C1qa^fl/fl^ homozygote males and females were a generous gift from Dr. Andrea Tenner (UC Irvine) (43). Ly6G^Cre/+^:C1qa^fl/fl^ mice were obtained by several backcrosses to C57BL/6J until Ly6G^Cre^/+ heterozygotes and C1qa^fl/fl^ homozygotes were achieved. Male mice between 8 and 12 weeks were used for experiments unless specified otherwise. The mice were housed under pathogen-free conditions at the University of Rochester Animal Facility. All mouse experiments were approved by the University Committee on Animal Resources (UCAR) at the University of Rochester.

### Mouse models of sepsis

Lipopolysaccharide (LPS)-induced endotoxemia and Cecal Ligation and Puncture (CLP) were performed according to Animal Resource Protocol approved by the Committee at the University of Rochester (UCAR 2008-039R). For endotoxemia assays, 8–12-week-old C57BL/6J, Ly6G^Cre/+^:C1qa^+/+^ (WT), or Ly6G^Cre/+^:C1qa^fl/fl^ (C1q cKO) male mice were weighed in order to deliver equal 12.5 mg/kg dose of LPS (*E. coli* O127:B8, Sigma-Aldrich) diluted in PBS *via* intraperitoneal (IP) injection. Animals were subsequently weighed daily for up to 7 days and closely monitored where in the event that percentage body weight loss exceeded 25% mice were euthanized *via* IACUC small rodent euthanasia protocols. For CLP, procedures were administrated following Rittirsch et al. (51). Briefly, mice were anesthetized with an IP injection of xylazine/ketamine cocktail (dose range of 80-100 mg/kg). Secondary anesthesia was maintained with Isoflurane/0_2_ gas mixture throughout the entire surgical procedure. Standard aseptic techniques were used to prepare the incision site. A vertical incision in the abdomen left lower quadrant was used to cut skin and peritoneum in order to access the peritoneal cavity. The cecum was ligated with silk sutures and punctured through with a 21-gauge needle, with sham mice receiving no ligation or puncture. Peritoneum and skin were approximated and closed using standard small animal surgical metal clips. Lidocaine was used topically on the wound site after stapling. Mice were resuscitated with 1 ml Ringers lactate injected subcutaneously. Animals were subsequently weighed daily for up to 7 days and given daily topical doses of Lidocaine until sacrifice to monitor disease progression and recovery. For antibody-mediated C1q neutralization *in vivo*, mice were pretreated with 10 μg of anti-C1q (clone JL-1, Hycult Biotech) or Rat IgG2b Isotype control (Abcam) in 100 μl of PBS *via* IP injection 1h prior to sepsis induction. For C1q treatment, mice were previously weighed and received single IP injection of LPS with or without treatment of 20 μg Native Human C1q protein (Abcam) administered as follows: 1 mg of C1q was reconstituted in 1 ml of distilled water with each animal receiving a single IP injection of 100 μl C1q/PBS (20 μl C1q + 80 μl PBS) at three separate time points. Dose 1 was given 30 min prior to LPS injection, dose 2 was given 6h after LPS injection and dose 3 was given 24h after LPS injection. Similar experimental processes were used for C3a (CompTech) and C5a (Sino Biological).

### Preparation of *ex vivo* single-cell suspensions from mouse tissues

For flow cytometry characterization of immune infiltrates in mice, the lungs, bone marrow (BM), spleen, kidneys, liver, peripheral blood, and peritoneal lavage isolates were removed from naive and septic mice at indicated time points and subsequently single-cell suspensions were prepared. Briefly, mice were lethally anesthetized with avertin (2-2-2-tribromoethanol, 240 mg/kg) intravenously (IV) and peripheral blood was collected *via* cardiac puncture. Mice were then perfused with 40 ml of cold PBS supplemented with 5 mM EDTA (Invitrogen). RBC lysis was performed using ACK lysing buffer (Invitrogen) in peripheral blood and spleen samples only. Leukocytes from all other organs were isolated by mincing tissues for 2.5 min and digested at 37°C with 80 U/ml Collagenase D (From *C. histolyticum*, Sigma-Aldrich) for 30 min. Cell-cell contacts were disrupted by adding 10 mM EDTA for 5 min following digestion. Tissue homogenates were achieved by forcing the suspensions 5-10 times through an 18-gauge needle and then passed through 70 µm filters. Single cell suspensions were then subjected to a room temperature 38% Percoll (GE Lifesciences) gradient for 30 min of centrifugation at 2000 rpm with no break. Leukocyte populations that spun to the bottom were then washed once before proceeding to staining.

### Sepsis patient sample

Patients admitted to Intensive Care Unit (ICU) with at least two out of four SIRS criteria (Temperature >38°C or <36°C, Heart rate >90/min, Respiratory rate >20 breaths/min or pCO_2_ <32mmHg, leukocyte count >12,000, < 4,000, or >10% immature forms on peripheral blood smear) and at least one acute organ dysfunction were enrolled into the study with a requirement that vital abnormalities were confirmed on two occasions (Demographics listed in **Table S1**). Additionally, we obtained clinical measurements, including scores for injury severity (APACHE II). The final diagnosis of sepsis was based on consensus review by three blinded expert clinicians who reviewed all clinical and severe sepsis was confirmed by clinical microbiological cultures. Blood samples were collected within 48h of diagnosis and 3-5 days later for a total of three collections. Severe sepsis was confirmed by clinical microbiological cultures. Lactate, Procalcitonin, Whole Blood Counts (WBC), and Absolute Neutrophil Counts (ANC) were determined by ICU laboratory bloodwork analysis. Blood samples were processed immediately for neutrophil isolation and split for flow cytometry, western blot cell lysates, and RNA for qRT-PCR. Serum was frozen immediately at -80°C and banked for cytokine assays.

### Human neutrophil isolation and stimulation

Blood was collected from healthy volunteers *via* antecubital vein puncture in heparin containing vacutainers. Granulocytes and erythrocytes were separated from whole blood by centrifugation through room temperature 1-step Polymorphs (Serumwerk Bernburg AG) density gradient at 1500 rpm for 45 min with no break. Remaining erythrocytes were removed by hypotonic lysis, yielding a neutrophil purity of > 98%. The Human Research Studies Review Board of the University of Rochester approved this study, and informed consent was obtained in accordance with the Declaration of Helsinki. To measure C1q expression and secretion, neutrophils were stimulated with either LPS (10 µg/ml) or fMLP (10 µM) in serum-free RPMI (Gibco) at 37°C for 1h.

### Flow cytometry and cell sorting

For mouse FACs analysis, Fc receptors were blocked with anti-CD16/32 (1:50, clone 93, eBioscience) for 10 min on ice. Samples were then stained for 15 min on ice with anti-CD45 (1:400, clone 30.F11, BD Biosciences) for leukocyte populations, anti-CD11b (1:400, clone M1/70, eBioscience) for myeloid cells, anti-Ly6G (1:200, clone 1A8, BioLegend) for neutrophil populations, anti-Ly6C (1:200, clone HK1.4, BioLegend) for monocytes, and anti-C1q (1:100, clone 7H8, Abcam) as indicated in the figure legends. For apoptosis staining, Annexin V (1:100, BD Pharmingen) was used per manufacturers guidelines. For neutrophil cell-sorting, cells were isolated from the BM and stained with anti-Ly6G, anti-mouse CD49c (1:100, polyclonal goat IgG, R&D Systems), and subsequently stained with secondary PE-conjugated donkey anti-goat IgG (Santa Cruz). DAPI (4′,6-diamidino-2-phenylindole, 1:30,000, BD Pharmingen) was added immediately before sample collection to distinguish live from dead cells. FACS-sorted CD49c^high^- and CD49c^low^-expressing mouse neutrophils from BM of endotoxemia-treated mice (bone marrow isolated 6 hrs after 34 mg/kg LPS (*E. coli* O55:B5, Sigma-Aldrich) injection) were resuspended in Buffer RLT (Qiagen) supplemented with β-mercaptoethanol and immediately frozen at -80°C for RNA-sequencing studies. For measuring surface expression on human neutrophils, purified mouse anti-human integrin α_3_/CD49c (1:100, clone P1B5, Millipore), anti-human CD64 (1:100, clone 32.2, Trillium Diagnostics), mouse IgG1 (1:100, eBioscience) isotype control and Phycoerythrin (PE) labeled Rat anti-mouse secondary antibodies were used. FACS-sorted CD49c^high^- and CD49c^low^-expressing human neutrophils were resuspended in Buffer RLT (Qiagen) supplemented with β-mercaptoethanol and immediately frozen at -80°C for RNA-sequencing studies. Samples were collected on an LSRII or Fortessa (BD) flow cytometer. Live neutrophils were sorted on a FACS Aria (BD) at the University of Rochester Flow Cytometry Core (FCC). Data were analyzed using Flow Jo software (TreeStar). For cell surface C1q staining, two million apoptotic human neutrophils were treated with C1q protein for 30 min at RT (250 ng/ml, 1% FBS + 1mM CaCl_2_ + HBS). After washing, cells were incubated with anti-C1q antibody (1 μg/ml) for 30 min at 4°C and then 1μg/ml of anti-rabbit IgG-PE for 30min at 4°C.

### RNA-sequencing

Total RNA was isolated using the RNeasy Plus Micro Kit (Qiagen) per manufacturer’s recommendations. RNA concentration was determined with the NanoDrop 1000 spectrophotometer (NanoDrop), and RNA quality assessed with the Agilent Bioanalyzer 2100 (Agilent). 1 ng of total RNA was pre-amplified with the SMARTer Ultra Low Input kit v4 (Clontech) per manufacturer’s recommendations. The quantity and quality of the subsequent cDNA was determined using the Qubit Fluorometer (Life Technologies) and the Agilent Bioanalyzer 2100. 150 pg of cDNA was used to generate Illumina compatible sequencing libraries with the NexteraXT library preparation kit (Illumina) per manufacturer’s protocols. The amplified libraries were hybridized to the Illumina single end flow cell and amplified using the cBot (Illumina). Single end reads of 100 nt were generated for each sample. Volcano plot analysis was done using GraphPad Prism v.7, and genes between conditions were compared based on adjusted p values and log2 fold change after filtering out genes with zero reads. Principle component analysis (PCA) plots and heat map representations were produced using RStudio (Version 1.3.1093, 2009-2020). Genes were clustered based on both Pearson and Spearman methods and plotted in terms of z-score. ‘ggplot2’, ‘pheatmap’, and ‘viridis’ libraries were installed and ‘heatmap.2’ or ‘pheatmap’ were used to represent data clustered using ‘viridis’ color palettes. The Enrichr gene ontology database was used to obtain categories of signaling pathways relating to genes that were downregulated and upregulated in isolated neutrophils. The listed categories were ranked by the likelihood that genes fall into the group as a function of *P* value. Bone marrow derived macrophage (BMDM) expression of C1q receptors under 0, 0.5, 1 and 2h LPS (100 ng/ml) stimulation was datamined using Gene Expression Omnibus (GEO) dataset GSE116220 (52).

### Mouse neutrophil and peritoneal macrophage isolation

Mouse neutrophils were isolated by negative selection (MoJoSort, BioLegend) from BM of naïve WT or C1q cKO 8-10-week-old male mice and allowed to senesce overnight in low-protein binding Eppendorf tubes in serum-free RPMI. FasL (0.2, 1, 5 μg/ml), fMLP (0.2, 1, 5 μM), or TNF-α (0.1, 1, 10 μg/ml) in serum-free RPMI at 37°C for 12hrs (Fig 4B). Peritoneal macrophages were isolated from WT mice. Briefly, cold PBS was injected into the peritoneum with a 30-gauge needle and peritoneal contents were gently massaged for 20-30 seconds. Peritoneal lavage was retrieved using a 21-gauge needle and spun down cells were cultured overnight on tissue-culture treated plates in complete RPMI supplemented with pen/strep, L-Glutamine, 10% heat-inactivated FBS, β-mercaptoethanol, nonessential amino acids, sodium pyruvate, and HEPES buffer at 37°C. Non-adherent cells were washed away 18-24hrs later and the majority of remaining adherent cells were macrophage. C1q receptor (C1qR) expression and macrophage phenotyping was done under the treatment of polarizing cytokines IFN-γ (40 ng/ml, Peprotech), IL-4 (1 μg/ml, Peprotech), as well as soluble C1q (10 μg/mL, Abcam), and plate-bound C1q (0.1 μg/mL, Abcam) pretreated for 1hr prior to cell culture. C1qR expression was quantified by flow cytometry using anti-CD11b (1:500, clone M1/70, BioLegend), anti-RAGE (1:400, clone 697023, R&D systems), anti-calreticulin (CRT, conc. 1:500, clone EPR3924, Abcam), anti-DC-SIGN (conc. 1:300, clone 902404, R&D systems), anti-SCARF-1 (conc. 1:500, clone 8578, Proteintech), and anti-LRP1 (CD91, conc 1: 300, clone EPR3724, Abcam). Macrophage polarization was measured as a percentage of F4/80+ cells (1:400, clone BM8, eBioscience) with varying expression levels of CD206 (1:200, clone C068C2, BioLegend), MerTK (1:200, clone 2B10C42, BioLegend), and CD86 (1:200, clone GL-1, BioLegend) as follows: M1 (CD45+/CD11b+/CD206^low^/MerTK^low^/CD86^high^) and M2 (CD45+/CD11b+/CD206^high^/MerTK^high^/CD86^low^).

### Phagocytosis assay

Apoptotic neutrophils were pre-stained with CypHer5E mono N-hydroxy succinimide (NHS) ester (GE Lifesciences) and subsequently cocultured with macrophages by briefly spinning in a non-tissue culture treated plate at 500 rpm for 1 min at a ratio of 1:10 macrophage: neutrophils for 1h. For human neutrophil phagocytosis assays, neutrophils were isolated from healthy donors and allowed to senesce overnight at 37°C. A human histiocytic lymphoma cell line (U937) was obtained from the American Type Culture Collection (ATCC) and maintained as nonadherent monocyte-like cells in suspension (53). U937s were stimulated with 2 nM Phorbol-12-Myristate-13-Acetate (PMA, Santa Cruz Technologies) overnight to induce macrophage differentiation and adherence. CypHer5E-stained apoptotic human neutrophils were cocultured with U937 macrophages at a ratio of 1:10 macrophage: neutrophils for 1hr in the presence of recombinant native human C1q (Abcam) at 10, 20, and 50 μg/ml. Cells were collected, and phagocytosis was measured by flow cytometry where CypHer5E absorbance and emission wavelengths are around 547 and 664 nm, respectively. CypHer5E has 2x greater absorbance and 5x brighter emission at pH 4.67 than at pH 9.15. Mean fluorescence intensity (MFI) of CypHer5E+ cells were calculated and analyzed using Flow Jo (Treestar) software. As a negative control, phagocytes were pretreated with Cytochalasin D (10 μM, Sigma-Aldrich) for 15 min prior to the engulfment assay.

### Neutrophil respiratory burst assay

WT and cKO neutrophils were isolated from BM using negative selection (see above) and subject to respiratory burst assay kit as per manufacturer’s instructions (Cayman Chemicals, Item # 601130). Briefly, 5 x 10^5^ neutrophils were cultured in polypropylene tubes, stimulated with PMA for 1hr at 37°C, stained with DHR-123, and analyzed on the LSRII flow cytometer.

### Western blotting and analysis

Mouse neutrophils isolated by negative selection from WT control or C1q cKO mice or human neutrophils were lysed with Pierce RIPA buffer (Thermo Scientific) supplemented with protease and phosphatase inhibitors (Thermo Scientific). Volumes of 6x SDS Laemmli were added directly and boiled at 95°C for 5 min. Total cell lysates were electrophoresed on SDS-polyacrylamide gel and transferred onto polyvinylidene difluoride (PVDF) membrane. The membrane was blocked with 5% milk in PBS containing 0.05% Tween 30 min to 1hr at room temperature and incubated with anti-C1q (Mouse: 1:50, clone 1151, rabbit anti-mouse, gift from Tenner lab at UC Irvine, or Human: 1:250, polyclonal rabbit anti-human, Dako) and shaken overnight at 4°C. The next day, membranes were incubated with HRP-anti-rabbit (Mouse) or HRP-anti-mouse (Human) IgG antibody (1:5000, Thermo scientific) for 1hr. Signals were visualized using Pico ECL substrate (Thermo Scientific). Loading controls were based on total β-actin directly conjugated to HRP (1:1000, Santa Cruz) added after stripping in 100 mM glycine-HCl pH 2.5, 150 mM NaCl, 0.1% Tween 20 for 30 min. Band intensities were quantified with Image J software.

### Immunofluorescence and bright-field microscopy

For “neutrophil C1q imaging”, human neutrophils were freshly isolated and placed in a glass-bottomed chamber (Millipore, Burlington, MA) coated with 10 µg/ml recombinant ICAM-1 and the indicated in L-15 medium (Thermo Fischer Scientific) at 37°C. For immunofluorescence microscopy, neutrophils bound to the glass slide were fixed with paraformaldehyde, permeabilized with 0.05% saponin and 0.05% Tween 20, and stained with the indicated antibodies (10 μg/ml C1q antibody + 2 μg/ml anti-rabbit alexa647 and 2 μg/ml FITC-conjugated anti-lactoferrin, -MMP9, and -MPO Abs for 2 hr). Microscopy was conducted using a TE2000-U microscope (Nikon, Melville, NY) and a 60x magnification objective. Migration analysis and image processing were performed using NIS (Nikon).

### Luminex assay and ELISAs

Human serum was collected from upper layer of 1-step polymorph and the levels of C1q were determined using Human Complement 1q ELISA kit (Abclonal). The expression of serum IL-6, IL-8 and TNF-α was quantified using MILLIPLEX MAP Human Cytokine/Chemokine Magnetic Bead Panel kit (Cat. # HCYTOMAG-60, EMD Millipore) according to manufacturer’s instructions. Briefly, patient’s serum samples were thawed on the ice at least one hour before the experiment. The standards or samples were added (in duplicate) into 96-well Luminex plate. Next, serum samples were mixed and incubated with antibody-linked magnetic beads at room temperature on a shaker (800 rpm) for 120 min and washed using BioPlex Pro magnetic wash station. Beads were resuspended in 125 µl of Bio-Plex assay buffer before reading on a calibrated Bio-Plex 200 system (Bio-Rad, Marnes-la-Coquette, France) and data were analyzed with Bio-Plex Manager 6.0 software. For mouse samples, serum and peritoneal lavage from individual mice were collected at 6 and 12hrs after LPS-induced endotoxemia and the levels of C1q were determined using Mouse Complement 1q ELISA kit (Abclonal). Cytokine levels of IL-6, IL-1β, and IL-10 were determined using DuoSet ELISA kits from R&D. For functional quantifications of C3a and C5a in the serum from CLP-induced septic mice, we used ELISA kit from ABclonal technology (Cat.# RK02648) and RayBio^®^ (Cat.#ELM-CCC5a) and followed the instructions. Streptavidin-HRP and TMB were purchased from Thermo Scientific. The colorimetric reaction was stopped with 2N sulfuric acid and measured at 450 nm.

### Real-time qPCR

Total RNA was prepared from human blood neutrophils using RNeasy Mini Kit. RNA (160 ng/reaction) was reverse transcribed using iScript cDNA Synthesis Kit (Bio-Rad), which contains pre-blended oligo (dT) and random primers. All cDNAs were divided into aliquots and stored at - 20°C until further use. Relative gene expression analysis was performed using optimized primers for C1qa, b, and c (**Table S2**) (54). cDNA was amplified using SsoAdvanced Universal SYBR Green Supermix (Bio-Rad) with CFX Connect realtime PCR detection system (Bio-Rad). Normalization between samples was performed using the average expression of three reference genes β-actin, SDHA, and TBP (**Table S2**) and relative quantification of gene expression was done using the 2^-ΔΔCT^ method (55).

### Histology and data analysis

Mice were euthanized as per UCAR protocol. Heart and lungs were carefully dissected out of the cavity and the heart and soft tissue were separated from both lungs using standard microsurgical dissecting techniques. Both lungs were placed over a sponge in fixing cassettes previously submerged in 70% ethanol in order to prevent organ folding during the fixation process. All cassettes were fixed in formalin for up to 2 days prior to histological processing. After 2 days, lungs were embedded in paraffin block and 4 μm sections were obtain using standard microtome techniques by the University of Rochester – Center for Musculoskeletal Research Histology core (CMSR) department. Four section levels at 50 μm apart were collected from the main airways. Paraffin slides were processed and stained with standard techniques for Hematoxylin & Eosin and Mason’s trichrome staining per the CMSR soft tissue protocols. All paraffin slides were digitally scanned using the CMSR – Histocore automated slide scanner (Olympus VS120 with VS-ASW v2.9.2) containing a brightfield camera with the following specifications: PIKE VC50 F505C and an Olympus U-TLU adapter 0.5x. Incorporated into the scanner there is an Olympus VS-BX microscope which contains the following specs: Mirror turret BX-RFAA, a Filter wheel ODB U-FFWO, and a UCB halogen lamp. All slides were scanned at the maximum magnification using the 40X objective (U PlanSApo 40X/0.95 ∞/0.11 – 0.23/FN26.5 UIS 2e). Focal points and areas were adjusted to incorporate all lung tissue in the slide. Tiff files were generated at 2x, 10x and 40x magnification and were analyzed using the VisioPharm and Image J software.

### Intravital microscopy

To visualize *in vivo* pulmonary microcirculation through a pulmonary imaging window, a custom-made video-rate laser scanning intravital microscopy system was implemented (IVM-CM, IVIM Technology Inc. South Korea) (28). Briefly, functional capillary imaging analysis was performed using a real-time video. Series of images were obtained after IV injection of anti-CD31 Alexa555 (33 μg for endothelium), anti-Ly6G-Alexa647 (25 μg for neutrophil), and FITC-2M (400 μg for blood flow). After splitting the colors in the video, sequential images of channels were processed by a median filter with a radius of two pixels to enhance the signal-to-noise ratio. A maximal intensity projection of 30–40 frames (1 frame/min) was generated. All image processing was performed by ImageJ. Image rendering with three-dimensional reconstruction, track analysis of neutrophils, and plotting track displacement was conducted using IMARIS 8.2 (Bitplane, Zurich, Switzerland).

### Bacterial load assay

Bacterial loads from the blood of IgG control and anti-C1q neutralizing antibody-treated septic mice were measured as described (19). In brief, after 48 h of post-CLP, mice were anesthetized using Avertin. Blood was collected by cardiac heart puncture. The collected blood was diluted in the ratios of 1:100, 1:1000, and 1:10000, and 100 μl. Each dilution was streaked on Tryptic Soy Agar (TSA) with 5% sheep blood plates (Remel). Plates were incubated under steady state conditions at 37°C with 5% CO2 for 24 h. Then bacterial colonies were enumerated for each animal and colony counts for each group were expressed as CFU/ml (n = 5/group).

### Statistical analysis

All statistical tests were performed with GraphPad Prism (v9). Statistical analysis was performed using Mantel-Cox test for survival curves, Two-way ANOVA, ordinary One-Way ANOVA with a Tukey’s multiple-comparison post-test, unpaired *t*-test, and Mann– Whitney test when appropriate. Differences were considered significant when *P* values were <0.05.

### Study approval

The Human Research Studies Review Board of the University of Rochester approved this study, and informed consent was obtained in accordance with the Declaration of Helsinki. All mouse procedures were approved by the University of Rochester Committee on Animal Resources under protocol UCAR-2008-039R and followed all Institutional Animal Care and Use Committee (IACUC) guidelines.

## Data availability statement

The original contributions presented in the study are included in the article/**supplementary material**, further inquiries can be directed to the corresponding author/s.

## Ethics statement

The studies involving human participants were reviewed and approved by Human Research Studies Review Board of the University of Rochester. The patients/participants provided their written informed consent to participate in this study.

## Author contributions

Conceptualization: AT and MiK; methodology: AT, RMo, RMa, KL, MaK, JSAP, CNP, and AP; investigation: AT, RMo, RMa, KL; funding acquisition: AT and MK; supervision: MK; writing – original draft: AT, RMo, AP, and MK. All authors contributed to the article and approved the submitted version.

## Funding

National Institutes of Health grant AI102851 (MK), National Institutes of Health grant AI149775 (MK), National Institutes of Health grant HL147525 (MK), National Institutes of Health grant HL160723 (MK, AP), National Institutes of Health grant T32AI118689 (AT).

## Acknowledgments

We thank E. Harrower for his technical assistance on the manuscript. We especially thank Dr. Andrea Tenner (UC Irvine) for C1q floxed mouse, Dr. Matthias Gunzer (University of Duisburg Essen) for Ly6G^Cre/Cre^ homozygote mouse, Dr. John Ashton for help with transcriptomic analyses and depositing RNA-sequencing data, Dr. Hongmei Yang for statistical data analyses, and all members of the Kim Laboratory for their comments during the course of these studies and input during preparation of the manuscript. We also thank Dr. Eunjoo Song and Dr. Pilhan Kim (KAIST, Korea) for their technical supports with the intravital imaging.

## Conflict of interest

The authors declare that the research was conducted in the absence of any commercial or financial relationships that could be construed as a potential conflict of interest.

## Publisher’s note

All claims expressed in this article are solely those of the authors and do not necessarily represent those of their affiliated organizations, or those of the publisher, the editors and the reviewers. Any product that may be evaluated in this article, or claim that may be made by its manufacturer, is not guaranteed or endorsed by the publisher.

## References

[1] Angus DC Linde-Zwirble WT Lidicker J Clermont G Carcillo J Pinsky MR. Epidemiology of severe sepsis in the united states: analysis of incidence, outcome, and associated costs of care. Crit Care Med (2001) 29:1303–10. doi: 10.1097/00003246-200107000-00002 11445675

[2] Liu V Escobar GJ Greene JD Soule J Whippy A Angus DC. Hospital deaths in patients with sepsis from 2 independent cohorts. JAMA (2014) 312:90–2. doi: 10.1001/jama.2014.5804 24838355

[3] Singer M Deutschman CS Seymour CW Shankar-Hari M Annane D Bauer M. The third international consensus definitions for sepsis and septic shock (Sepsis-3). JAMA (2016) 315:801–10. doi: 10.1001/jama.2016.0287 PMC4968574 26903338

[4] Kaukonen KM Bailey M Pilcher D Cooper DJ Bellomo R. Systemic inflammatory response syndrome criteria in defining severe sepsis. N Engl J Med (2015) 372:1629–38. doi: 10.1056/NEJMoa1415236 25776936

[5] Sweeney TE Perumal TM Henao R Nichols M Howrylak JA Choi AM. A community approach to mortality prediction in sepsis *via* gene expression analysis. Nat Commun (2018) 9:694. doi: 10.1038/s41467-018-03078-2 29449546 PMC5814463

[6] Stanski NL Wong HR. Prognostic and predictive enrichment in sepsis. Nat Rev Nephrol (2020) 16:20–31. doi: 10.1038/s41581-019-0199-3 31511662 PMC7097452

[7] Nuytinck HK Offermans XJ Kubat K Goris JA. Whole-body inflammation in trauma patients. an autopsy study. Arch Surg (1988) 123:1519–24. doi: 10.1001/archsurg.1988.01400360089016 3056336

[8] van der Poll T van de Veerdonk FL Scicluna BP Netea MG. The immunopathology of sepsis and potential therapeutic targets. Nat Rev Immunol (2017) 17:407–20. doi: 10.1038/nri.2017.36 28436424

[9] Brown KA Treacher DF. Neutrophils as potential therapeutic targets in sepsis. Discov Med (2006) 6:118–22. 17234146

[10] Brown KA Brain SD Pearson JD Edgeworth JD Lewis SM Treacher DF. Neutrophils in development of multiple organ failure in sepsis. Lancet (2006) 368:157–69. doi: 10.1016/S0140-6736(06)69005-3 16829300

[11] Nathan C Ding A. Nonresolving inflammation. Cell (2010) 140:871–82. doi: 10.1016/j.cell.2010.02.029 20303877

[12] Soehnlein O. Multiple roles for neutrophils in atherosclerosis. Circ Res (2012) 110:875–88. doi: 10.1161/CIRCRESAHA.111.257535 22427325

[13] Luo HR Loison F. Constitutive neutrophil apoptosis: mechanisms and regulation. Am J Hematol (2008) 83:288–95. doi: 10.1002/ajh.21078 17924549

[14] Matute-Bello G Liles WC Radella F 2nd Steinberg KP Ruzinski JT Jonas M. Neutrophil apoptosis in the acute respiratory distress syndrome. Am J Respir Crit Care Med (1997) 156:1969–77. doi: 10.1164/ajrccm.156.6.96-12081 9412582

[15] Garlichs CD Eskafi S Cicha I Schmeisser A Walzog B Raaz D. Delay of neutrophil apoptosis in acute coronary syndromes. J Leukoc Biol (2004) 75:828–35. doi: 10.1189/jlb.0703358 14742636

[16] Wong SH Francis N Chahal H Raza K Salmon M Scheel-Toellner D. Lactoferrin is a survival factor for neutrophils in rheumatoid synovial fluid. Rheumatology (Oxford) (2009) 48:39–44. doi: 10.1093/rheumatology/ken412 19029133 PMC2639483

[17] Bagaitkar J Huang J Zeng MY Pech NK Monlish DA Perez-Zapata LJ. NADPH oxidase activation regulates apoptotic neutrophil clearance by murine macrophages. Blood (2018) 131:2367–78. doi: 10.1182/blood-2017-09-809004 PMC5969376 29618478

[18] Fialkow L Fochesatto Filho L Bozzetti MC Milani AR Rodrigues Filho EM Ladniuk RM. Neutrophil apoptosis: a marker of disease severity in sepsis and sepsis-induced acute respiratory distress syndrome. Crit Care (Lond Engl) (2006) 10:R155. doi: 10.1186/cc5090 PMC1794458 17092345

[19] Lerman YV Lim K Hyun YM Falkner KL Yang H Pietropaoli AP. Sepsis lethality *via* exacerbated tissue infiltration and TLR-induced cytokine production by neutrophils is integrin alpha3beta1-dependent. Blood (2014) 124:3515–23. doi: 10.1182/blood-2014-01-552943 PMC4256905 25278585

[20] Vassallo G Newton RW Chieng SE Haeney MR Shabani A Arkwright PD. Clinical variability and characteristic autoantibody profile in primary C1q complement deficiency. Rheumatology (Oxford) (2007) 46:1612–4. doi: 10.1093/rheumatology/kem207 17890276

[21] Thielens NM Tedesco F Bohlson SS Gaboriaud C Tenner AJ. C1q: A fresh look upon an old molecule. Mol Immunol (2017) 89:73–83. doi: 10.1016/j.molimm.2017.05.025 28601358 PMC5582005

[22] Mukherjee S Cabrera MA Boyadjieva NI Berger G Rousseau B Sarkar DK. Alcohol increases exosome release from microglia to promote complement C1q-induced cellular death of proopiomelanocortin neurons in the hypothalamus in a rat model of fetal alcohol spectrum disorders. J Neurosci (2020) 40:7965–79. doi: 10.1523/JNEUROSCI.0284-20.2020 PMC7548688 32887744

[23] Botto M Dell'Agnola C Bygrave AE Thompson EM Cook HT Petry F. Homozygous C1q deficiency causes glomerulonephritis associated with multiple apoptotic bodies. Nat Genet (1998) 19:56–9. doi: 10.1038/ng0598-56 9590289

[24] Hasenberg A Hasenberg M Mann L Neumann F Borkenstein L Stecher M. Catchup: a mouse model for imaging-based tracking and modulation of neutrophil granulocytes. Nat Methods (2015) 12:445–52. doi: 10.1038/nmeth.3322 25775045

[25] Paidassi H Tacnet-Delorme P Garlatti V Darnault C Ghebrehiwet B Gaboriaud C. C1q binds phosphatidylserine and likely acts as a multiligand-bridging molecule in apoptotic cell recognition. J Immunol (2008) 180:2329–38. doi: 10.4049/jimmunol.180.4.2329 PMC2632962 18250442

[26] Fraser DA Laust AK Nelson EL Tenner AJ. C1q differentially modulates phagocytosis and cytokine responses during ingestion of apoptotic cells by human monocytes, macrophages, and dendritic cells. J Immunol (2009) 183:6175–85. doi: 10.4049/jimmunol.0902232 PMC2843563 19864605

[27] Elliott MR Ravichandran KS. The dynamics of apoptotic cell clearance. Dev Cell (2016) 38:147–60. doi: 10.1016/j.devcel.2016.06.029 PMC4966906 27459067

[28] Park I Kim M Choe K Song E Seo H Hwang Y. Neutrophils disturb pulmonary microcirculation in sepsis-induced acute lung injury. Eur Respir J (2019) 53. doi: 10.1183/13993003.00786-2018 PMC6437604 30635296

[29] Soehnlein O Lindbom L. Phagocyte partnership during the onset and resolution of inflammation. Nat Rev (2010) 10:427–39. doi: 10.1038/nri2779 20498669

[30] Wynn TA Vannella KM. Macrophages in tissue repair, regeneration, and fibrosis. Immunity (2016) 44:450–62. doi: 10.1016/j.immuni.2016.02.015 PMC4794754 26982353

[31] Li K Sacks SH Zhou W. The relative importance of local and systemic complement production in ischaemia, transplantation and other pathologies. Mol Immunol (2007) 44:3866–74. doi: 10.1016/j.molimm.2007.06.006 17768105

[32] van Schaarenburg RA Magro-Checa C Bakker JA Teng YK Bajema IM Huizinga TW. C1q deficiency and neuropsychiatric systemic lupus erythematosus. Front Immunol (2016) 7:647. doi: 10.3389/fimmu.2016.00647 28082982 PMC5186770

[33] Walport MJ Davies KA Botto M. C1q and systemic lupus erythematosus. Immunobiology (1998) 199:265–85. doi: 10.1016/S0171-2985(98)80032-6 9777411

[34] Cortes-Hernandez J Fossati-Jimack L Petry F Loos M Izui S Walport MJ. Restoration of C1q levels by bone marrow transplantation attenuates autoimmune disease associated with C1q deficiency in mice. Eur J Immunol (2004) 34:3713–22. doi: 10.1002/eji.200425616 15517607

[35] Zoghi S Ziaee V Hirschmugl T Jimenez-Heredia R Krolo A Boztug K. Exome sequencing revealed C1Q homozygous mutation in pediatric systemic lupus erythematosus. Allergol Immunopathol (Madr) (2018) 46:594–8. doi: 10.1016/j.aller.2018.02.004 29739689

[36] Botto M. Links between complement deficiency and apoptosis. Arthritis Res (2001) 3:207–10. doi: 10.1186/ar301 PMC128896 11438036

[37] Luo B Wang J Liu Z Shen Z Shi R Liu YQ. Phagocyte respiratory burst activates macrophage erythropoietin signalling to promote acute inflammation resolution. Nat Commun (2016) 7:12177. doi: 10.1038/ncomms12177 27397585 PMC4942576

[38] N AG Quintana JA Garcia-Silva S Mazariegos M Gonzalez de la Aleja A Nicolas-Avila JA. Phagocytosis imprints heterogeneity in tissue-resident macrophages. J Exp Med (2017) 214:1281–96. doi: 10.1084/jem.20161375 PMC5413334 28432199

[39] Woo MS Yang J Beltran C Cho S. Cell surface CD36 protein in Monocyte/Macrophage contributes to phagocytosis during the resolution phase of ischemic stroke in mice. J Biol Chem (2016) 291:23654–61. doi: 10.1074/jbc.M116.750018 PMC5095418 27646002

[40] Fox S Ryan KA Berger AH Petro K Das S Crowe SE. The role of C1q in recognition of apoptotic epithelial cells and inflammatory cytokine production by phagocytes during helicobacter pylori infection. J Inflamm (Lond) (2015) 12:51. doi: 10.1186/s12950-015-0098-8 26357509 PMC4563842

[41] Cai Y Teo BH Yeo JG Lu J. C1q protein binds to the apoptotic nucleolus and causes C1 protease degradation of nucleolar proteins. J Biol Chem (2015) 290:22570–80. doi: 10.1074/jbc.M115.670661 PMC4566231 26231209

[42] Gyorffy BA Kun J Torok G Bulyaki E Borhegyi Z Gulyassy P. Local apoptotic-like mechanisms underlie complement-mediated synaptic pruning. Proc Natl Acad Sci U S A (2018) 115:6303–8. doi: 10.1073/pnas.1722613115 PMC6004452 29844190

[43] Fonseca MI Chu SH Hernandez MX Fang MJ Modarresi L Selvan P. Cell-specific deletion of C1qa identifies microglia as the dominant source of C1q in mouse brain. J Neuroinflamm (2017) 14:48. doi: 10.1186/s12974-017-0814-9 PMC5340039 28264694

[44] Lubbers R van Essen MF van Kooten C Trouw LA. Production of complement components by cells of the immune system. Clin Exp Immunol (2017) 188:183–94. doi: 10.1111/cei.12952 PMC5383442 28249350

[45] Armbrust T Nordmann B Kreissig M Ramadori G. C1Q synthesis by tissue mononuclear phagocytes from normal and from damaged rat liver: up-regulation by dexamethasone, down-regulation by interferon gamma, and lipopolysaccharide. Hepatology (1997) 26:98–106. doi: 10.1053/jhep.1997.v26.pm0009214457 9214457

[46] Zhang D Frenette PS. Cross talk between neutrophils and the microbiota. Blood (2019) 133:2168–77. doi: 10.1182/blood-2018-11-844555 PMC6524562 30898860

[47] Castillo DJ Rifkin RF Cowan DA Potgieter M. The healthy human blood microbiome: Fact or fiction? Front Cell Infect Microbiol (2019) 9:148. doi: 10.3389/fcimb.2019.00148 31139578 PMC6519389

[48] Gosiewski T Ludwig-Galezowska AH Huminska K Sroka-Oleksiak A Radkowski P Salamon D. Comprehensive detection and identification of bacterial DNA in the blood of patients with sepsis and healthy volunteers using next-generation sequencing method - the observation of DNAemia. Eur J Clin Microbiol Infect Dis (2017) 36:329–36. doi: 10.1007/s10096-016-2805-7 PMC5253159 27771780

[49] Nikkari S McLaughlin IJ Bi W Dodge DE Relman DA. Does blood of healthy subjects contain bacterial ribosomal DNA? J Clin Microbiol (2001) 39:1956–9. doi: 10.1128/JCM.39.5.1956-1959.2001 PMC88056 11326021

[50] Paisse S Valle C Servant F Courtney M Burcelin R Amar J. Comprehensive description of blood microbiome from healthy donors assessed by 16S targeted metagenomic sequencing. Transfusion (2016) 56:1138–47. doi: 10.1111/trf.13477 26865079

[51] Rittirsch D Huber-Lang MS Flierl MA Ward PA. Immunodesign of experimental sepsis by cecal ligation and puncture. Nat Protoc (2009) 4:31–6. doi: 10.1038/nprot.2008.214 PMC2754226 19131954

[52] McNab FW Ewbank J Rajsbaum R Stavropoulos E Martirosyan A Redford PS. TPL-2-ERK1/2 signaling promotes host resistance against intracellular bacterial infection by negative regulation of type I IFN production. J Immunol (2013) 191:1732–43. doi: 10.4049/jimmunol.1300146 PMC3796877 23842752

[53] King CH Fields BS Shotts EB Jr. White EH. Effects of cytochalasin d and methylamine on intracellular growth of legionella pneumophila in amoebae and human monocyte-like cells. Infect Immun (1991) 59:758–63. doi: 10.1128/iai.59.3.758-763.1991 PMC258324 1997428

[54] Agostinis C Bulla R Tripodo C Gismondi A Stabile H Bossi F. An alternative role of C1q in cell migration and tissue remodeling: contribution to trophoblast invasion and placental development. J Immunol (2010) 185:4420–9. doi: 10.4049/jimmunol.0903215 20810993

[55] Livak KJ Schmittgen TD. Analysis of relative gene expression data using real-time quantitative PCR and the 2(-delta delta C(T)) method. Methods (2001) 25:402–8. doi: 10.1006/meth.2001.1262 11846609

