# Supplementary material for: Neutrophil heterogeneity in complement C1q expression associated with sepsis mortality
Source: Front Immunol. 2022 Aug 2;13:965305. doi: 10.3389/fimmu.2022.965305 (PMC9380571; doi:10.3389/fimmu.2022.965305)
Supplement: Supplementary file 1 [file DataSheet_1.pdf]

Supplementary Figure 1

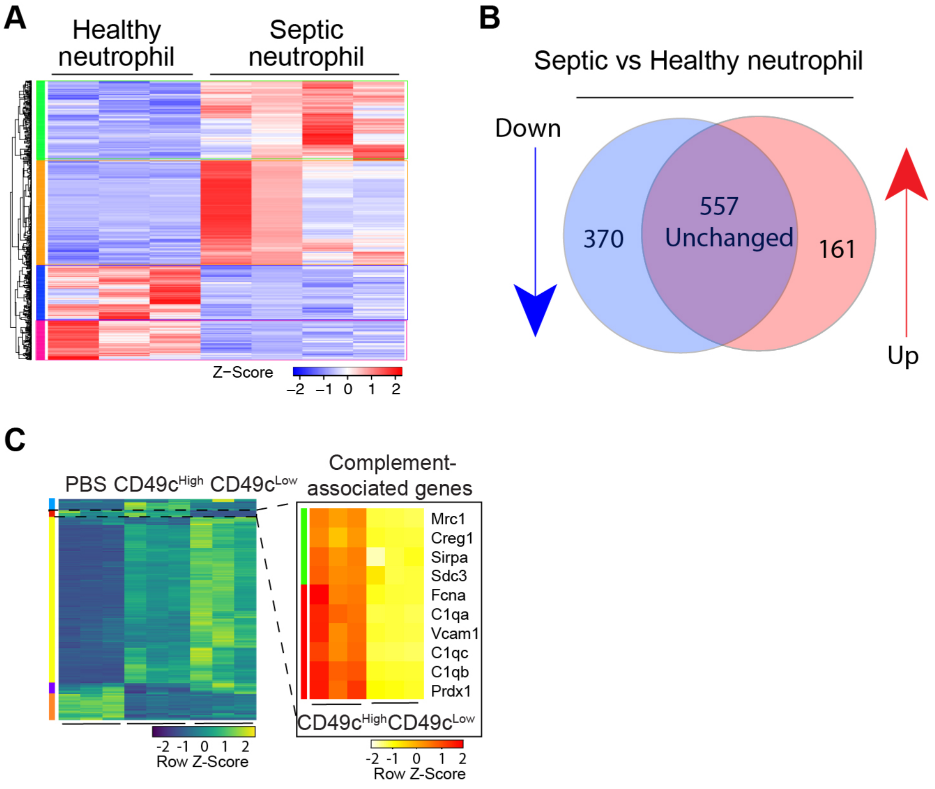

Fig. S1.

Septic patient neutrophils display elevated levels of CD49c and altered gene signatures.

a, Heatmap plotting RNA-sequencing of healthy subjects (n=3) vs. septic patients (n=4) representing top 0.25% significant genes differentially regulated during sepsis. **b,** Venn diagram indicating three groups of genes either downregulated (blue), upregulated (red), or unchanged (purple, overlap). **v**, Heatmap of gene expressions (z-score), *left*. The top 326 genes chosen from highest 0.25% p-value summary. *Right*: Highlighted complement-associated gene category '2' of CD49c^{high} versus CD49c^{low} neutrophils (n=3 per group).

Supplementary Figure 2

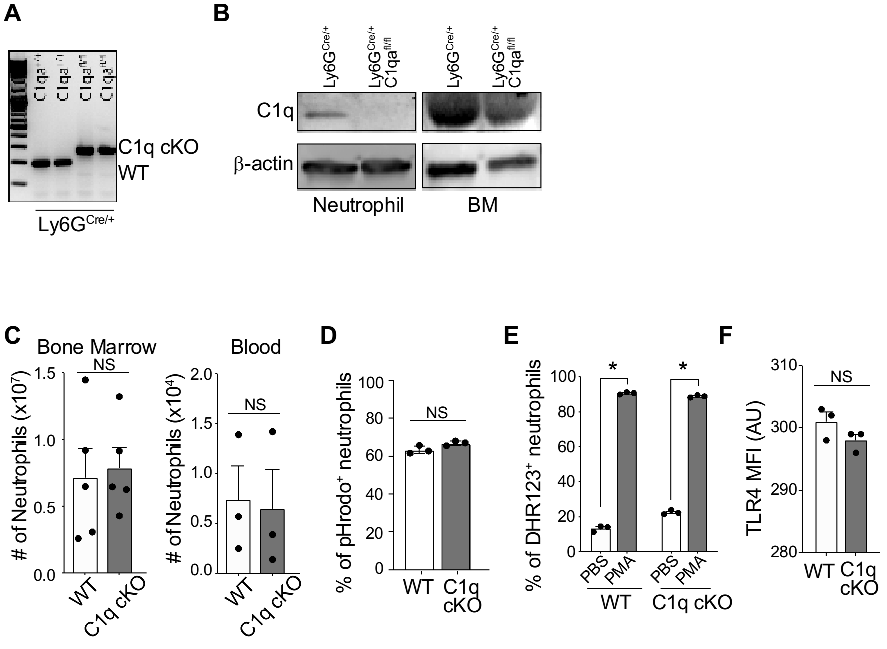

Fig. S2.

Neutrophil specific Cre-recombinase driven by Ly6G promoter successfully floxes out the C1qa gene.

a, DNA agarose gel of n=2 mice with either unfloxed C1qa alleles (WT band at 171 bps) or floxed C1qa alleles (C1q cKO floxed band at 261 bps) in neutrophils. **b**, Western blot of C1q protein from neutrophils isolated from the bone marrow of WT or C1q cKO mice. **c**, C1q cKO mice show normal neutrophil counts in the bone marrow and blood. **d**, Flow cytometry analysis of uptake of opsonized *E. coli* pHrodo particles in WT vs. C1q cKO neutrophils (n = 3 per group). **e**, Respiratory burst assay of WT vs C1q cKO neutrophils stimulated with PMA show no differences in neutrophil antimicrobial activity (n=3 per group). **f,** Baseline TLR4 surface expression of WT vs C1q cKO neutrophils (n=3 per group). Data are presented as mean ± SEM and analyzed by student's t-test for **c, d,** and **f** and ordinary one-way ANOVA with Tukey's multiple comparison post-test in **e**.

Supplementary Figure 3

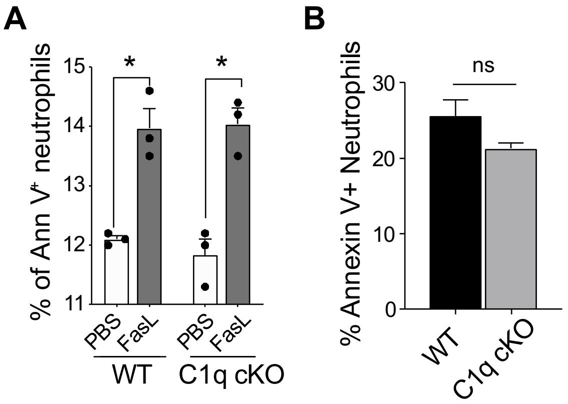

Fig. S3.

C1q cKO neutrophils undergo similar amounts of apoptosis *in vivo*

a, *C1qa* deficient neutrophils undergo similar levels of apoptosis when stimulated with 100 ng/ml of FasL for 1h *in vitro*. Quantified as percentage of total CD45+/CD11b+/Ly6G+ cells, n=3 per group. **b,** Annexin V expression in neutrophils isolated from BM of WT or KO mice of 8-week-old mice, n=3-4 per group. NS= Not significant. Data are presented as mean ± SEM and analyzed by ordinary one-way ANOVA with Tukey's multiple comparison post-test in **a** and student's t-test for **b**. (*P<0.05).

Table S1.

Summary of human subject descriptors for healthy and sepsis groups

Age, SOFA and APACHE II scores are represented as mean ± SD

	Healthy	Sepsis
Number	14	123
Male	67%	50.6%
Female	33%	49.4%
Survived	-	82.1%
Deceased	-	17.9%
Age	45.8 ± 14.0	60.53 ± 15.59
APACHE II score	-	23.08 ± 8.29
SOFA score	-	6.12 ± 3.84
Condition / infection source	-	Blood, Urine, Pulmonary, Skin/catheter, Intra-abdominal
Positive culture	-	51%
Gram negative infection	-	20%
Gram positive infection	-	66.7%
Gram negative and Gram positive infection	-	13.3%

Table S2.

Real-time qPCR primers

Sample	Primers	Sequence (5'-3')
C1qa	Forward	ATGGTGACCGAGGACTTGTG
	Reverse	GTCCTTGATGTTTCCTGGGC
C1qb	Forward	ACCCCAGGGATAAAAGGAGAG
	Reverse	GGCAGAGAAGGCGATTTTCTG
C1qc	Forward	AAGGATGGGTACGACGGACTG
	Reverse	TTTCTGCTTGTATCTGCCCTC
C2	Forward	GTG GAC TGG AGA GAA CTG AAT G
	Reverse	TTC AAA GAC CTG GTG CAG AG
C3	Forward	CCC AAC CTC AGC TAC ATC ATC
	Reverse	GAG GTC CTG GCA TTG TTT CT
C4	Forward	GGA GAC ATC TAA CTG GCT TCT G
	Reverse	CCC TGC ATG CTC CTA TGT ATC
C5	Forward	CGA TGG AGC CTG CGT TAA TA
	Reverse	CTT GCG ACG ACA CAA CAT TC
C6	Forward	GTG GCT ATG ACA CCT GCT ATG
	Reverse	GAG TTG GTT TCC ACC CTT GA
C7	Forward	GTG CGA GGA AGA AGG GTT TAG
	Reverse	CCT TAT GCT GGT GAC AGA GAT G
C8	Forward	TAG CCT GTG AGG TCT CCT ATC
	Reverse	GTC TTG TCT TAC GTC TTC CAG AG
C9	Forward	TGC AGA GAC AGA GTG GTA GA
	Reverse	CCG GTT ACA GAG TCC ATT GTA G
β-actin	Forward	CATCGAGCACGGCATCGTCA

	Reverse	TAGCACAGCCTCCATAGCAAC
SDHA	Forward	TGGGAACAAGAGGGCATCTG
	Reverse	CCACCACTGCATCAAATTCATG
TCP	Forward	TGCCCGAAACGCCGCATATAATC
	Reverse	GTGTGGACTGTTCTTCACTCTTGG

Movie S1.

Vascular neutrophil in sepsis-induced ALI model (PBS). FITC Dextran (blue) was injected by the tail vein of the naive mouse (PBS), and intravital pulmonary imaging was performed to visualize neutrophils (anti-Ly6G) in the pulmonary capillaries (anti-CD31). Scale bar, 50 μm and time is marked as minutes.

Movie S2.

Vascular neutrophil in sepsis-induced ALI model (LPS). FITC Dextran (blue) was injected by the tail vein of the sepsis mouse (LPS), and intravital pulmonary imaging was performed to visualize neutrophil accumulation (anti-Ly6G) in the pulmonary capillaries (anti-CD31) 18 h after LPS stimulation. Scale bar, 50 μm and time is marked as minutes.

Movie S3.

Vascular neutrophil aggregates in sepsis-induced ALI model (LPS). FITC Dextran (blue) was injected by the tail vein of the sepsis mouse (LPS), and intravital pulmonary imaging was

performed to visualize neutrophil accumulation (anti-Ly6G) in the pulmonary capillaries (anti-CD31) 18 h after LPS stimulation. Scale bar, 50 μm and time is marked as minutes.

Movie S4.

Vascular neutrophil in sepsis-induced ALI model (LPS+C1q). FITC Dextran (blue) was injected by the tail vein of the sepsis mouse (LPS), and intravital pulmonary imaging was performed to visualize neutrophil accumulation (anti-Ly6G) in the pulmonary capillaries (anti-CD31) 18 h after LPS stimulation. C1q protein (50 μg/50 μl) was IV injected at -18 h, -12 h, and 0 h of imaging. Scale bar, 50 μm and time is marked as minutes.
